# Implementation and integration of a multidisciplinary pharmacogenomics service in an underserved integrated behavioral health clinic

**DOI:** 10.3389/fphar.2025.1594032

**Published:** 2025-05-22

**Authors:** Bader M. Alghamdi, Sara Rogers, Susan Roberman, Meredith Williamson, Ladan Panahi

**Affiliations:** ^1^ Department of Clinical Pharmacy, Albaha College of Pharmacy, Albaha University, Al Bahah, Saudi Arabia; ^2^ Department of Pharmacy Practice, Irma Lerma Rangel School of Pharmacy, Texas A&M University, Kingsville, TX, United States; ^3^ Department of Translational Medical Sciences, School of Medicine, Texas A&M Health Science Center, Bryan, TX, United States; ^4^ American Society of Pharmacovigilance, Houston, TX, United States; ^5^ Department of Primary Care and Rural Medicine, Texas A&M Health Science Center School of Medicine, Bryan, TX, United States

**Keywords:** PGx implementation, ambulatory care pharmacy practice, clinical service, pharmacogenomic, pharmacy practice

## Abstract

**Objective:**

To assess the feasibility and impact of incorporating a multidisciplinary pharmacogenomics (PGx) service within an underserved behavioral health clinic, with an emphasis on clinician perceptions.

**Methodology:**

This study was conducted in two phases at the Texas A&M Family Care Clinic. Phase one involved an online cross-sectional survey of the multidisciplinary clinic team to assess their knowledge, attitudes, and readiness for PGx integration. Phase two detailed the development and implementation of a PGx service within the Integrated Behavioral Health (IBH) clinic, outlining the workflow and collaborative approach used to offer genetic testing to eligible patients.

**Key findings:**

Of the 23 survey participants, 91% believed the PGx service would positively impact patient care, and 87% expressed interest in receiving PGx-related training. Confidence in pharmacists’ ability to lead the service was reported by 65% of respondents. The primary concerns identified included cost of care, clinical utility, and potential workflow disruptions. A collaborative implementation model was developed, including preemptive and reactive testing pathways.

**Conclusion:**

The implementation of a pharmacist-driven PGx service in an underserved behavioral health clinic was well-received by the clinical team and deemed feasible. While concerns regarding resources and workflow were noted, strong interest in training and multidisciplinary collaboration highlights the potential for scalable PGx service models in similar settings.

## 1 Introduction

Management of mental health disorders presents many challenges, including issues related to the effectiveness of treatments and the presence of side effects. For example, selective serotonin reuptake inhibitors (SSRIs) are widely prescribed for depression; however, their effectiveness in reducing baseline symptoms varies from 40% to 60%, with remission rates ranging from 30% to 45%, and up to one-third of patients experiencing recurrent depression symptoms during treatment (Agency for Healthcare Research and Quality, 2023). Genetic factors play a role in how individuals respond to psychotropic medications, alongside other elements such as drug-drug interactions, dietary influences, pathophysiological conditions, and environmental factors (Zierhut et al., 2017). Many of these variables can be identified and addressed through patient discussions. However, genetic influences specifically require targeted genetic testing to be accurately determined.

Pharmacogenomics offers potential solutions to challenges in psychiatric treatment (Wang et al., 2023; Oslin et al., 2022; Van Westrhenen et al., 2020). Many antidepressant and antipsychotic medications are metabolized by cytochrome P450 enzymes, particularly CYP2D6 and CYP2C19. Genetic variations in these enzymes can lead to altered drug metabolism, resulting in subtherapeutic effects or increased toxicity, including heightened side effects or poor response (Taylor et al., 2020). Consequently, differing statuses in metabolizing these enzymes can result in varying levels of the drug in the body and different reactions to treatment (Jameson et al., 2021). By understanding the relationship between genetic variants and drug metabolism personalized dosing can be optimized, potentially improving treatment outcomes, and reducing adverse events. Pharmacogenomics has demonstrated clinical utility in psychiatry by reducing adverse drug reactions and optimizing antidepressant selection, thereby enhancing treatment efficacy and patient safety (Ahmed et al., 2024).

Underserved populations often face disparities in access to mental healthcare, limited treatment personalization, and higher risks of adverse medication effects. These challenges are compounded by structural barriers such as limited provider availability, lower health literacy, and socioeconomic factors (Hodgkinson et al., 2017; Arredondo et al., 2023). In their study on perceptions of pharmacogenetic testing among underserved patients, Gawronski et al. found that while overall awareness of PGx was limited, there was strong interest in its potential benefits. Participants expressed openness to PGx-guided care, particularly when it could reduce medication trial-and-error and improve safety. The authors emphasized the need for equitable PGx implementation strategies, noting that underserved patients are both in need of and receptive to personalized approaches when barriers such as cost, education, and provider support are addressed (Gawronski et al., 2022). These findings underscore the importance of integrating PGx in safety-net health systems and behavioral health clinics that serve medically underserved populations.

## 2 Methods

### 2.1 Setting and participants

#### 2.1.1 Setting information

Our research was conducted in Texas A&M Family Care Clinic (TAMU-FCC), a primary care clinic located in Bryan, Texas, a suburban area with a diverse population of approximately 87,792 residents and serving the Brazos Valley Region with a population of 379,734 residents (United States Census Bureau, 2024). The clinic serves a wide range of patients, including low-income families and immigrant communities, providing services in both English and Spanish to accommodate the diverse patient demographic.

TAMU-FCC offers a range of medical services, including preventive medicine, chronic disease management, acute care, pediatrics, obstetrics, sports medicine, multiple procedures, and mental health services. The Integrated Behavioral Health (IBH) clinic, serving underserved populations, aims to improve outcomes and reduce costs by providing accessible behavioral health services integrated with primary care (Miller-Matero et al., 2016). The clinic employs a team-based care approach involving healthcare professionals such as psychiatrists, psychologists, therapists, case managers, social workers, medical residents, pharmacists, and primary care physicians to deliver comprehensive care.

### 2.2 Phase one, family care clinic team survey feedback

A cross-sectional, online Likert-scale survey was conducted from October 2023 to November 2023 targeting the entire Texas A&M Family Care Clinic team. The survey involved 23 participants: 9 physicians, 6 family medicine residents, 2 psychiatry residents, 2 behavioral health consultants, 1 psychologist, 1 nurse practitioner, 1 licensed clinical social worker, and 1 clinical psychology student. Regarding referrals to IBH clinic, only physicians and nurse practitioners providing care in the TAMU-FCC system have the authority to refer patients. Out of the respondents, 6 were active members of the IBH clinic, while 15 were not part of the IBH clinic actively refer patients. Additionally, 1 participant is neither affiliated with the IBH clinic nor has referral privileges, and 1 participant chose not to answer this question. The survey consisted of 15 questions covering demographic information, knowledge assessment, attitudes, practices, and included open-ended items for additional feedback. It was administered electronically via REDCap, utilizing a secure online platform. Participants were assured of the confidentiality of their responses and informed of the voluntary nature of their participation. The study protocol was reviewed and approved by the Texas A&M institutional review board (IRB2023-1087M).

### 2.3 Phase two, development of an integrated PGx service

**FIGURE 1:**
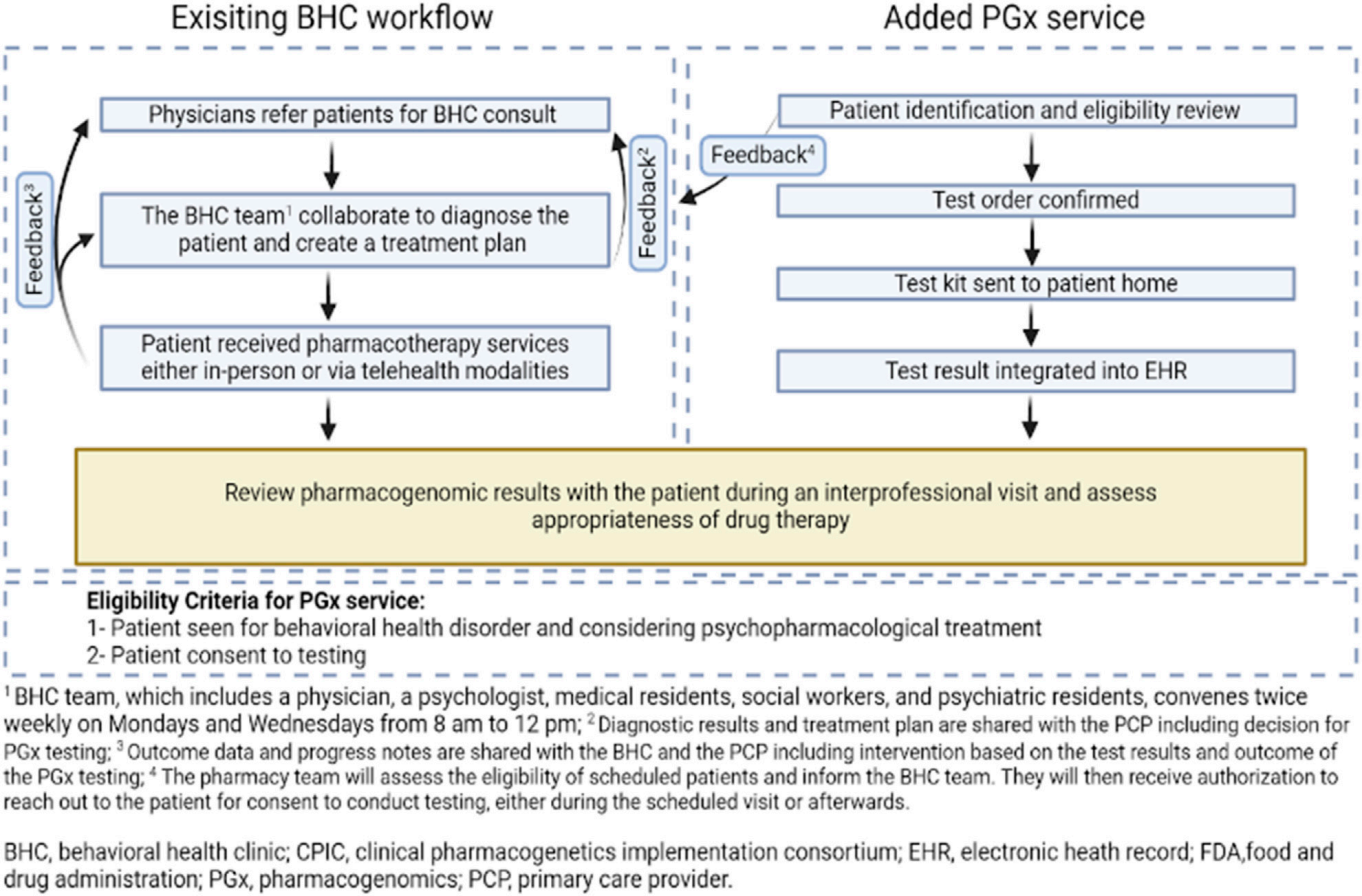
Workflow map integrating pharmacogenomics into IBH, aiming to provide patients with the most tailored and effective treatment based on their genetic makeup.

In November 2023, following the survey administration, the initial steps in designing and implementing a clinical PGx service were undertaken. Communication began afterward regarding the usefulness of clinical PGx testing to the clinician involved in direct patient care. ​The possibility of offering this service was discussed at multidisciplinary meetings which led to the development of the workflow map integrating PGx service into the behavioral health clinic (Figure 1).

Pharmacogenomic testing was offered as part of a multidisciplinary service within the behavioral health clinic. Eligible patients were adults (aged 18 years or older) who were actively receiving care from the behavioral health team and being considered for psychopharmacologic treatment. Testing was contingent upon informed consent, after which the care team determined whether the patient was a candidate for reactive testing or preemptive testing.

Saliva-based genetic testing kits were mailed to patients for at-home self-collection. Results were typically available within 7–10 business days and were accessed by the clinical pharmacist through a secure, HIPAA-compliant online platform provided by the testing laboratory. The test panel included both pharmacokinetic gene variants (e.g., CYP2D6, CYP2C19, CYP1A2, CYP3A4, CYP3A5) and pharmacodynamic or transporter-related genes (e.g., SLC6A4, HTR2A) relevant to psychiatric medication response.

Once results were available, the pharmacist reviewed the report and collaborated with the prescribing provider to interpret the findings. PGx-informed recommendations, such as medication selection or dose adjustments, were then discussed during multidisciplinary team meetings and incorporated into the patient’s treatment plan. Clinical follow-up allowed for ongoing assessment of treatment outcomes based on the recommendations.

## 3 Results

### 3.1 Family care clinic feedback

#### 3.1.1 Provider awareness and perceptions of PGx

Approximately half of respondents (n = 12, 52%) reported having a good or excellent understanding of the role of pharmacogenomics in the treatment of mental health conditions. A strong majority (n = 21, 91%) believed that implementing a pharmacist-driven PGx service would positively impact patient care. Confidence in pharmacists’ ability to lead PGx services was expressed by 15 respondents (65%) (Table 1).

**TABLE 1:** Survey respondent attitudes and awareness toward Implementation and Integration of a Multidisciplinary Driven Pharmacogenomics Service in an Underserved Integrated Behavioral Health Clinic.

Survey question	Category	Response description	N (%)
Knowledge of PGx role	Understanding of PGx Role	12 respondents indicated ‘good’ or ‘excellent’ understanding	12 (52.17%)
		11 Respondents selected other options including neutral, negative, or unsure responses	11 (47.83%)
Perceived impact on care	Perception of Impact	21 respondents indicated a ‘positive impact'	21 (91.3%)
		2 Respondents selected other options including neutral, negative, or unsure responses	2 (8.7%)
Confidence in pharmacists	Confidence in Pharmacists	15 respondents indicated ‘confidence'	15 (65.22%)
		8 Respondents selected other options including neutral, negative, or unsure responses	8 (34.78%)
Enhancement of treatment plans	Treatment Plans	21 respondents indicated PGx would ‘enhance treatment plans'	21 (91.3%)
		2 Respondents selected other options including neutral, negative, or unsure responses	2 (8.7%)
Importance of collaboration	Multidisciplinary Collaboration	14 respondents rated collaboration as ‘extremely important'	14 (60.87%)
		9 Respondents selected other options including neutral, negative, or unsure responses	9 (39.13%)
Interest in PGx training	Interest in PGx Training	20 respondents showed ‘interest in training'	20 (86.96%)
		3 Respondents selected other options including neutral, negative, or unsure responses	3 (13.04%)
Concerns related to PGx implementation	Costs of PGx Care	11 respondents selected ‘cost’ as a concern	11 (47.83%)
	Clinical Utility and Evidence Base	8 respondents selected ‘clinical utility’ as a concern	8 (34.78%)
	Workflow Disruptions	6 respondents selected ‘workflow disruptions'	6 (26.09%)
	Other concerns (staffing, visits, knowledge)	3 respondent each mentioned need for staffing, visits, and pharmacist’s psychiatric knowledge	3 (13.04%)

#### 3.1.2 Perceived benefits and collaboration needs

The majority of respondents (n = 21, 91%) felt that PGx would enhance personalized treatment plans. Furthermore, 14 respondents (61%) rated multidisciplinary collaboration as “extremely important” for the service’s success. Interest in receiving PGx-related training or educational sessions was reported by 20 respondents (87%) (Table 1).

#### 3.1.3 Reported barriers to implementation

The most frequently cited concerns were cost of care (n = 11, 48%), clinical utility and evidence base (n = 8, 35%), and potential disruptions to clinic workflow (n = 6, 26%). Isolated individual concerns included the need for additional staffing (n = 1), more patient visits (n = 1), and pharmacist knowledge of psychiatric conditions (n = 1) (Table 1).

### 3.2 Development of an integrated PGx service

As a result of the collaborative efforts between the behavioral health team and the pharmacy team, two approaches for implementing PGx testing were identified: reactive and preemptive testing. Reactive testing is performed in those who exhibit adverse drug reactions or treatment resistance, guiding a more tailored therapeutic strategy (Relling et al., 2010). Preemptive testing involves assessing genetic markers before the start of therapy to anticipate drug responses and tailor treatments proactively (Keeling et al., 2019; Rogers, 2023). The existing BHC workflow starts with physicians referring patients for BHC consultations. The added PGx service begins with the pharmacy team identifying patients for testing (Figure 1). Once a patient is identified, consent is obtained during the BH visit, the test is ordered, and a kit is sent to the patient’s home. The results, typically processed within 2 weeks, are then integrated into the Electronic Health Record (EHR) and discussed in subsequent provider visits. The impact of the test results on treatment is assessed, and a collaborative care plan is established during a follow-up visit with the multidisciplinary team. Patients continue to be monitored regularly, allowing for the evaluation of outcomes based on the tailored treatment plans influenced by the PGx testing insights. Clinical outcome data, such as treatment response or reduction in side effects were not quantitatively assessed at this stage.

## 4 Discussion

This implementation study aimed to assess the feasibility of introducing a pharmacist-driven pharmacogenomics (PGx) service in an underserved behavioral health setting. The positive perception of PGx among the multidisciplinary team aligns with findings from previous studies highlighting the clinical potential of PGx to guide psychotropic medication selection and reduce treatment failures (Yau, et al., 2015).

Our findings resonate with the conclusions drawn by Bradley et al., who reported that PGx-guided antidepressant selection can reduce adverse drug reactions and improve the timeliness of achieving symptom remission (Bradley et al., 2018). Although our study did not assess treatment outcomes directly, the high interest in PGx training and the team’s confidence in pharmacist leadership suggest a readiness to adopt such innovations, echoing the supportive attitudes described in larger reviews of PGx implementation.

Notably, our study contributes to the growing evidence supporting PGx integration in primary care and mental health settings, particularly those serving populations with limited access to personalized care. Unlike many PGx implementation efforts situated in academic medical centers, this project took place in a community-based integrated care clinic, providing practical insights into resource-constrained settings (Bousman et al., 2021).

However, several limitations must be acknowledged. First, the data are descriptive and based on a small sample from a single clinic, which limits generalizability. Second, while provider attitudes and workflow structures were assessed, patient outcome data were not collected. Future studies should incorporate clinical endpoints such as symptom improvement, medication adherence, or adverse event reduction. Additionally, PGx test results were not integrated into the Electronic Health Record (EHR), which could hinder seamless implementation. Finally, concerns around cost, test interpretation, and staffing highlight that feasibility does not equate to ease of adoption.

Ethical considerations also played an essential role in the implementation of PGx testing. Prior to testing, patients participated in brief counseling sessions delivered by the pharmacist or behavioral health provider. These sessions covered the purpose of testing, the types of genes analyzed, potential implications for medication management, and privacy protections. Patients were clearly informed that participation was voluntary, and informed consent was obtained prior to sample collection. Test results were accessed through a secure, HIPAA-compliant online platform provided by the testing laboratory and were not stored in the local electronic health record. Patients retained the right to decline or withdraw from PGx-guided care at any time.

Despite these limitations, the survey used in Phase I primarily served as a readiness assessment to inform the design of a PGx service tailored to this specific clinic. Our findings suggest that even in underserved clinics, there is an appetite for PGx services, particularly when championed by pharmacists and supported through interprofessional collaboration (Norris et al., 2023). These results may inform future studies that seek to evaluate clinical outcomes and explore models for sustainable PGx service delivery in resource-limited behavioral health environments.

## 5 Conclusion

PGx offers significant potential for enhancing psychiatric pharmacotherapy, particularly in underserved populations. The findings from this early implementation project highlight a positive perception of PGx among behavioral health providers and demonstrate the feasibility of embedding pharmacist-led PGx services within a multidisciplinary team. However, for successful broader implementation, several challenges must be addressed—namely, the financial burden of testing, the need for comprehensive clinician education, and the accessibility of PGx resources for patients and providers. Future work should focus on evaluating patient outcomes, integrating PGx into clinical workflows, and exploring scalable models of care in diverse settings.

## Data Availability

The raw data supporting the conclusions of this article will be made available by the authors, without undue reservation.
