# Supplementary material for: Implementation and integration of a multidisciplinary pharmacogenomics service in an underserved integrated behavioral health clinic
Source: Front Pharmacol. 2025 May 22;16:1594032. doi: 10.3389/fphar.2025.1594032 (PMC12142042; doi:10.3389/fphar.2025.1594032)
Supplement: Supplementary file 1 [file DataSheet1.pdf]

This survey aims to gauge the team's perceptions, concerns, and willingness to collaborate with the pharmacist in the context of a pharmacogenomics service. Adjustments can be made based on specific needs or feedback.

Q1. By checking "I agree to participate in this survey," you are agreeing to participate in the survey and that your responses to this and subsequent surveys will be used for evaluating the pharmacist-driven pharmacogenomics service.

1. I agree to participate in this survey.
2. I do NOT agree to participate in this survey.

Q2. Which of the following best describes your current role on the interdisciplinary behavioral health team?

1. Social worker
2. Medical assistant
3. Psychologist
4. Physician
5. Family medicine resident
6. Psychiatry resident
7. Other

Q3. If you chose “other” for the above question, please type below what your current role is on the interdisciplinary behavioral health team.

Free Text

Q4. How would you describe your knowledge pertaining to the role of pharmacogenomics in the treatment of mental health conditions?

1. I can confidently explain this concept.
2. I have a good understanding of this topic.
3. I have heard of this but don't know the details.
4. I am not sure about this.
5. I have no knowledge of this topic.

Q5. How do you perceive the ***potential impact*** of a pharmacist-driven pharmacogenomics service on patient care in the behavioral health clinic?

1. Extremely positive impact
2. Moderately positive impact
3. Neutral/no impact
4. Moderately negative impact
5. Extremely negative impact
6. I am not sure

Q6. How confident are you in the ability of pharmacists to provide pharmacogenomics services in the context of behavioral health?

1. Extremely confident

2. Moderately confident
3. Neutral
4. Moderately unconfident
5. Extremely unconfident
6. I am not sure

Q7. What ***concerns***, if any, do you have about the integration of a pharmacist-driven pharmacogenomics service in a behavioral health clinic? (Select all that apply)

1. Potential for increased costs
2. Patient privacy and data security
3. Clinical utility and evidence base
4. Workflow disruptions
5. I don't have concerns
6. I am not sure
7. Other

Q8. If you chose "other" for the above question, please type below your concerns about the integration of a pharmacist-driven pharmacogenomics service in a behavioral health clinic.

Free Text

Q9. How do you anticipate the ***response of patients*** to the introduction of a pharmacist-driven pharmacogenomics service?

1. Extremely positive
2. Moderately positive
3. Neutral/no opinion
4. Moderately negative
5. Extremely negative
6. I am not sure

Q10. How do you perceive the potential of a pharmacist-driven pharmacogenomics service in ***improving medication efficacy*** for patients of the behavioral health clinic?

1. Extremely beneficial
2. Moderately beneficial
3. Neutral/no perceived benefit
4. Moderately detrimental
5. Extremely detrimental
6. I am not sure

Q11. Do you believe that the integration of a pharmacist-driven pharmacogenomics service will lead to a reduction in ***medication-related adverse events***?

1. Strongly agree
2. Agree
3. Neutral/undecided
4. Disagree

5. Strongly disagree
6. I am not sure

Q12. How do you perceive the potential of a pharmacist-driven pharmacogenomics service in ***enhancing*** personalized treatment plans for patients?

1. Extremely beneficial
2. Moderately beneficial
3. Neutral/no perceived benefit
4. Moderately detrimental
5. Extremely detrimental
6. I am not sure

Q13. Do you believe that the pharmacist-driven pharmacogenomics service will provide valuable insights into ***drug-drug interactions*** specific to individual genetic profiles?

1. Strongly agree
2. Agree
3. Neutral/undecided
4. Disagree
5. Strongly disagree
6. I am not sure

Q14. In your opinion, how important is multidisciplinary collaboration in ensuring the success of the pharmacist-driven pharmacogenomics service?

1. Extremely important
2. Moderately important
3. Neutral
4. Moderately unimportant
5. Not important at all
6. I am not sure

Q15. Would you be interested in receiving training or educational sessions on pharmacogenomics to better collaborate with the pharmacist?

1. Definitely yes
2. Probably yes
3. Neutral/undecided
4. Probably no
5. Definitely no
6. I am not sure

Thank you for taking the time to complete this survey. Your insights and feedback are invaluable to us as we are integrating a pharmacogenomics service at the behavioral health clinic. We believe in the power of multidisciplinary collaboration and your perspectives will play a crucial role in shaping the future of patient care at the clinic. We appreciate your dedication to improving patient outcomes and look forward to working together to achieve our shared goals.
